# Laboratory-based versus non-laboratory-based World Health Organization risk equations for assessment of cardiovascular disease risk

**DOI:** 10.1186/s12874-023-01961-1

**Published:** 2023-06-15

**Authors:** Azizallah Dehghan, Ali Rayatinejad, Rozhan Khezri, Dagfinn Aune, Fatemeh Rezaei

**Affiliations:** 1 Noncommunicable Diseases Research Center, Fasa University of Medical Sciences, Fasa, Iran; 2 Student Research Committee, Jahrom University of Medical Sciences, Jahrom, Iran; 3 Department of Epidemiology, School of Public Health, Iran University of Medical Sciences, Tehran, Iran; 4 Department of Epidemiology and Biostatistics, School of Public Health, Imperial College London, London, UK; 5 Department of Nutrition, Oslo New University College, Oslo, Norway; 6 Research Center for Social Determinants of Health, Jahrom University of Medical Sciences, Jahrom, Iran

**Keywords:** Laboratory-based, Non-laboratory-based, WHO, Cardiovascular disease, Risk prediction, Sensitivity, Specificity

## Abstract

**Background:**

The WHO model has laboratory-based and non-laboratory-based versions for 10-year risk prediction of cardiovascular diseases. Due to the fact that in some settings, there may not be the necessary facilities for risk assessment with a laboratory-based model, the present study aimed to determine the agreement between laboratory-based and non-laboratory-based WHO cardiovascular risk equations.

**Methods:**

In this cross-sectional study, we used the baseline data of 6796 individuals without a history of cardiovascular disease and stroke who participated in the Fasa cohort study. The risk factors of the laboratory-based model included age, sex, systolic blood pressure (SBP), diabetes, smoking and total cholesterol, while the non-laboratory-based model included age, sex, SBP, smoking and BMI. Kappa coefficients was used to determine the agreement between the grouped risk and Bland–Altman plots were used to determine the agreement between the scores of the two models. Sensitivity and specificity of non-laboratory-based model were measured at the high-risk threshold.

**Results:**

In the whole population, the agreement between the grouped risk of the two models was substantial (percent agreement = 79.0%, kappa = 0.68). The agreement was better in males than in females. A substantial agreement was observed in all males (percent agreement = 79.8%, kappa = 0.70) and males < 60 years old (percent agreement = 79.9%, kappa = 0.67). The agreement in males ≥ 60 years old was moderate (percent agreement = 79.7%, kappa = 0.59). The agreement among females was also substantial (percent agreement = 78.3%, kappa = 0.66). The agreement for females < 60 years old, (percent agreement = 78.8%, kappa = 0.61) was substantial and for females ≥ 60 years old, (percent agreement = 75.8%, kappa = 0.46) was moderate. According to Bland–Altman plots, the limit of agreement was (95%CI: -4.2% to 4.3%) for males and (95%CI: -4.1% to 4.6%) for females. The range of agreement was suitable for both males < 60 years (95%CI: -3.8% to 4.0%) and females < 60 years (95%CI: -3.6% to 3.9%). However, it was not suitable for males ≥ 60 years (95% CI: -5.8% to 5.5%) and females ≥ 60 years (95%CI: -5.7% to 7.4%). At the high-risk threshold of 20% in non-laboratory and laboratory-based models, the sensitivity of the non-laboratory-based model was 25.7%, 70.7%, 35.7%, and 35.4% for males < 60 years, males ≥ 60 years, females < 60 years, and females ≥ 60 years, respectively. At the high-risk threshold of 10% in non-laboratory-based and 20% in laboratory-based models, the non-laboratory model has high sensitivity of 100% for males ≥ 60 years, females < 60 years, females ≥ 60 years, and 91.4% for males < 60 years.

**Conclusion:**

A good agreement was observed between laboratory-based and non-laboratory-based versions of the WHO risk model. Also, at the risk threshold of 10% to detect high-risk individuals, the non-laboratory-based model has acceptable sensitivity for practical risk assessment and the screening programs in settings where resources are limited and people do not have access to laboratory tests.

## Background

Cardiovascular Diseases (CVDs) account for one third of deaths worldwide and are the leading causes of mortality globally. In the last decade, the number of deaths caused by CVDs in the world has increased by 12.5% globally [1]. CVDs affect most populations around the world, at all income levels, but the highest overall burden of CVDs is currently in low- and middle-income countries (LMICs), where, due to lack of financial and human resources, deaths from CVDs occurs at a younger age than in high-income countries [2, 3]. In LMICs, including the Eastern Mediterranean Region (EMR), 50% of deaths and 80% of the global burden of CVDs occur [4]. It has been predicted that the prevalence of CVDs in Iran will increase sharply due to several reasons, including an aging population, unhealthy diets, inadequate physical activity, and smoking, and in the near future the burden of CVDs will reach 45.4% to 72% [5–7].

Hypertension, diabetes, obesity, smoking and high serum cholesterol are the most important risk factors for CVDs, which together with socio-economic factors, inactivity, and unhealthy diets have a synergistic effect on the occurrence of CVDs. The important thing is that these risk factors can be modified and controlled with effective interventions [8].

One important way to reduce the burden of CVDs is to identify individuals at high risk and intervene using appropriate management protocols [9]. Many global guidelines recommend CVD risk assessment charts to identify individuals at risk of CVD. The purpose of risk-based management is to carry out specific preventive and therapeutic interventions [10, 11]. One of the methods for assessing the 10-year risk of CVDs is the WHO risk model, which is used to predict the 10-year risk of fatal and non-fatal cardiovascular outcomes. The first WHO risk charts were presented in 2007, and in 2019, WHO updated the old CVDs risk assessment charts.

The WHO CVD Risk Chart Working Group was assembled to promote the development of updated models for predicting CVD risks better suited to the needs of LMICs. While the previous charts provide estimates for 14 WHO regions, the new charts produce estimates for 21 Institute for Health Metrics and Evaluation (IHME) Global Burden of Disease (GBD) regions [12].

In the revised WHO 2019 model, the previous laboratory-based risk assessment models were modified and new non-laboratory-based (office-based or BMI-based) models were presented. In the laboratory-based WHO cardiovascular risk equation, the factors age, gender, smoking status, SBP, serum total cholesterol and diabetes status are used to predict the 10-year risk of fatal and non-fatal cardiovascular outcomes, while the non-laboratory-based WHO cardiovascular risk equation, include age, sex, smoking status, SBP and BMI [12]. WHO laboratory-based and non-laboratory-based cardiovascular risk equations have been developed for various global regions and confirmed in cohort studies [13, 14]. Efforts have been made to develop non-laboratory-based models that can predict the risk of CVDs as accurately as laboratory-based algorithms with even more flexibility [12].

In Iran, the CVD mortality rates have been increasing, in contrast to high-income countries in Europe and North America [15]. For this reason, it is necessary to estimate the 10-year risk of CVDs according to risk algorithms. Several studies have evaluated the 10-year risk of CVDs in Iran. In one study, the 10-year risk was estimated with laboratory-based WHO cardiovascular risk equations [16] and in others, the agreement between laboratory-based and non-laboratory-based Framingham and Globorisk cardiovascular risk scores was evaluated [17, 18].

According to the 2021 World Bank report, Iran is a low to middle income country [19]. In Iran, no specific method for predicting the 10-year risk of CVDs has been established, therefore models provided in other countries or models provided by WHO have been used to estimate the risk of CVDs. In addition, most risk prediction equations require blood glucose levels and lipid profile, which can make risk assessment in resource-poor settings very costly or impractical [20]. Considering that in LMICs, there may not be enough laboratory facilities in the centers where basic health services are provided, or people may not be able to pay for or have access to laboratory tests, non-laboratory-based models are more practical for risk estimation. The present study was conducted with the aim of evaluating the agreement between laboratory-based and non-laboratory-based WHO cardiovascular risk equations in a large population using Bland–Altman method and kappa statistic.

## Methods

### Fasa cohort study design

This cross-sectional study was conducted using the Fasa cohort study baseline data. The Fasa cohort study is a part of the PERSIAN cohort study, which was designed and proceeded in 2014 and included 18 different ethnic and geographic groups in 18 provinces of Iran [21]. The Fasa cohort study has been described in detail in a previous publication [22]. In brief, it was conducted on 10,138 individuals aged ≥ 35 years in the rural area of Sheshdeh in Fasa city in southern Iran. Data were collected between 2015 and 2016 with the help of trained interviewers who were native residents. This was an advantage as they could interact with people more effectively in providing the information they needed. The participants’ demographic characteristics (age, gender, education, occupation, etc.), disease history (diabetes, hypertension, cardiovascular diseases, stroke, cancer, etc.) and smoking history (cigarette, hookah, and pipe) were collected. Anthropometric characteristics (height, weight and waist circumference) as well as blood pressure (BP) were measured. Moreover, blood samples were taken for biochemical tests. All measurements in this study were performed according to the guidelines of the PERSIAN cohort study [21]. In this study, individuals < 40 years old and > 74 years old (2152 people), along with individuals who had a history of CVDs or stroke (1189 people), were excluded from the study. Overall, 6796 individuals aged 40–74 years without a history of CVDs or stroke were included in the analysis.

### CVD risk

The 10-year risk of CVDs was calculated using WHO laboratory-based and non-laboratory-based cardiovascular risk prediction equations. The details have been published elsewhere [23]. The laboratory-based model included the variables age, gender, smoking, diabetes, SBP and total cholesterol, while the non-laboratory-based model included age, sex, smoking, SBP and BMI [12].

A smoker as someone who had smoked ≥ 100 cigarettes in their lifetime, and a current smoker as someone who smoked either every day or some days at the time of the study. Subjects were interviewed about their smoking status and after 12 h of fasting, blood samples were taken for biochemical tests including blood sugar and cholesterol. Moreover, diabetes status was evaluated by the previous history of the disease, drug history or fasting blood sugar (FBS) ≥ 126 mg/dL. BP was measured twice, with a 15-min interval (after 15 min of rest), and the mean SBP and diastolic blood pressure (DBP) were recorded. Hypertension was defined as SBP of ≥ 140 mmHg or DBP of ≥ 90 mmHg, or being on antihypertensive medication. Lastly, BMI was obtained by dividing weight by height squared (kg/m2).

### Statistical analysis

For the categorical variables, numbers and percentages, and for the continuous ones, means and standard deviations (SDs) were calculated. The 10-year CVD risk was calculated with laboratory-based and non-laboratory-based CVD risk equations. Details regarding the analysis have been published before [17, 23]. The agreement between laboratory-based and non-laboratory-based WHO versions was determined by type of risk (quantitative and qualitative). Percent agreement and kappa statistics were used for categorical risk and Bland–Altman plots were used for risk scores.

Bland–Altman plots were used to estimate continuous agreement scores between the two models. Details of this method have been presented before [17], and here, we report it, briefly. Using this method, the risk difference between laboratory-based and non-laboratory-based models (laboratory-based minus non-laboratory-based) and also the mean scores (laboratory-based + non-laboratory-based) /2) of these two models were calculated. Bland–Altman plots were plotted by sex and age group (< 60 and ≥ 60 years). Mean risk score differences and 95% confidence intervals (CIs) were estimated using paired t-tests. For each person, the Bland–Altman plot shows the difference between two scores versus the average of two scores. The average of two scores is shown on the horizontal axis and the difference between the two scores on the vertical axis in the Bland–Altman plots. Since the true risk of CVD cannot be determined for each individual, then the best estimates available would be the average scores of laboratory-based and non-laboratory-based models [24]. Also, 95% of the limits of agreement range is shown by the average difference of the scores ± 1.96 SD of the difference of the scores.

In the WHO risk models, risk scores are divided in five groups. Risk scores < 5%, 5% to < 10%, 10% to < 20%, 20% to < 30%, and ≥ 30% indicated a very low-, low-, moderate-, high-, and very high- risk groups, respectively [12]. The concordance between two models was calculated by percent agreement and kappa statistics. The kappa coefficient is classified into six groups: poor (≤ 0), slight (0.01–0.20), fair (0.21–0.40), moderate (0.41–0.60), substantial (0.61–0.80), and almost perfect (0.81–1.0) [25].

Given that the first step of the screening program goes through the non-laboratory model and through this filter, high-risk people will be selected for further laboratory evaluations assuming the laboratory model as the gold standard. In order to define high-risk people based on the models, the risk threshold of 20% was considered as it has been shown to have good sensitivity for intensive care in high-risk individuals [26]. The traditional performance indices (sensitivity, specificity, positive predictive value, and negative predictive value) were measured. Moreover, the risk threshold of 10% was considered for the non-laboratory model to achieve a reasonable sensitivity [27]. Also, the Matthews correlation coefficient (MCC) was estimated to calculate the quality of binary classifications. MCC generates a high score only if the binary predictor is able to predict the majority of the positive data samples and the majority of negative data samples correctly. MCC is between -1 (perfect misclassification) and + 1 (perfect classification). While MCC = 0 means the classifier performance is no better than random classification [28].

Statistical analyses were performed with Statistical Package for Social Science (IBM SPSS Statistics for Windows, Version 23.0. Armonk, NY: IBM Corp) and Stata Statistical Software (Stata 14 for windows, Stata Corp., College Station, TX, USA). *P*-values less than 0.05 were considered statistically significant.

### Ethical considerations

This study was approved by the Ethics Committee of Jahrom University of Medical Sciences (IR.JUMS.REC.1401.095). The data were collected anonymously and each participant signed informed consent forms.

## Results

In this study, 6796 individuals with the average age of 51.0 ± 7.8 years participated and 46.5% of the participants were males. In Table 1 a summary of the distribution and means of risk factors is shown. The prevalence of smoking was higher among males than females (40.0% vs. 2.4%). However, the prevalence of hypertension and diabetes was higher in females. Moreover, the means of SBP, DBP, cholesterol and BMI were higher in females than in males. The mean 10-year CVD risk was higher in the laboratory-based model than in the non-laboratory-based model (7.4 ± 5.3 vs. 7.2 ± 4.9). In both models, the mean 10-year risk of CVD was higher in males compared to females, in a way that the mean laboratory-based CVD risk scores were 7.9 ± 5.6 in males and 6.9 ± 5.1 in females, and the mean non-laboratory-based CVD risk scores were 7.8 ± 5.4 in males and 6.6 ± 4.4 in females (Table 2).

**Table 1:** Participant characteristics among 6796 adults in Fasa cohort study

Variables	Males (*n* = 3157)	Females (*n* = 3639)	Total (*n* = 6796)
	**N (%)**	**N (%)**	**N (%)**
**Age range (years)**			
< 60	2594(82.2)	3032(83.3)	5626(82.8)
≥ 60	563(17.8)	607(16.7)	1170(17.2)
**Smoking (now)**	1264(40.0)	86(2.4)	1350(19.9)
**Diabetes**	251(8.0)	621(17.1)	872(12.8)
**Hypertension**	325(10.3)	964(26.5)	1289(19.0)
**DBP (**Mean mmHg ± SD)	74.6 ± 11.7	75.4 ± 11.9	75.0 ± 11.8
**SBP** (Mean mmHg ± SD)	111.2 ± 17.5	113.4 ± 19.1	112.4 ± 18.4
**HDL** (Mean mmol/l ± SD)	1.2 ± 0.4	1.4 ± 0.4	1.3 ± 0.4
**Chol** (Mean mmol/l ± SD)	4.7 ± 0.9	5.0 ± 1.0	4.9 ± 1.0
**BMI** (kg/m^2^), (Mean ± SD)	24.2 ± 4.5	26.8 ± 4.8	25.6 ± 4.8

*DBP* Diastolic blood pressure, *SBP* Systolic blood pressure, *HDL* High density lipoprotein, *Chol* Cholesterol, *BMI* body mass index

**Table 2:** CVDs risk scores according to Laboratory-based and non-laboratory-based equations among 6796 adults in Fasa cohort study

CVDs risk model	Total (*n* = 6796)	Males (*n* = 3157)	Females (*n* = 3639)	*p*-value
**Laboratory-based CVDs risk score** (10- year, %), (Mean ± SD)				
< 60 years old	5.8 ± 3.8	6.3 ± 4.0	5.4 ± 3.4	< 0.001
≥ 60 years old	14.9 ± 5.4	15.3 ± 5.5	14.5 ± 5.2	0.008
Total	7.4 ± 5.3	7.9 ± 5.6	6.9 ± 5.1	< 0.001
**Non-laboratory-based CVD risk score** (10- year, %), (Mean ± SD)				
< 60 years old	5.7 ± 3.2	6.2 ± 3.6	5.2 ± 2.8	< 0.001
≥ 60 years old	14.5 ± 5.0	15.5 ± 5.7	13.7 ± 4. 1	< 0.001
Total	7.2 + 4.9	7.8 ± 5.4	6.6 ± 4.4	< 0.001

**Fig. 1:**
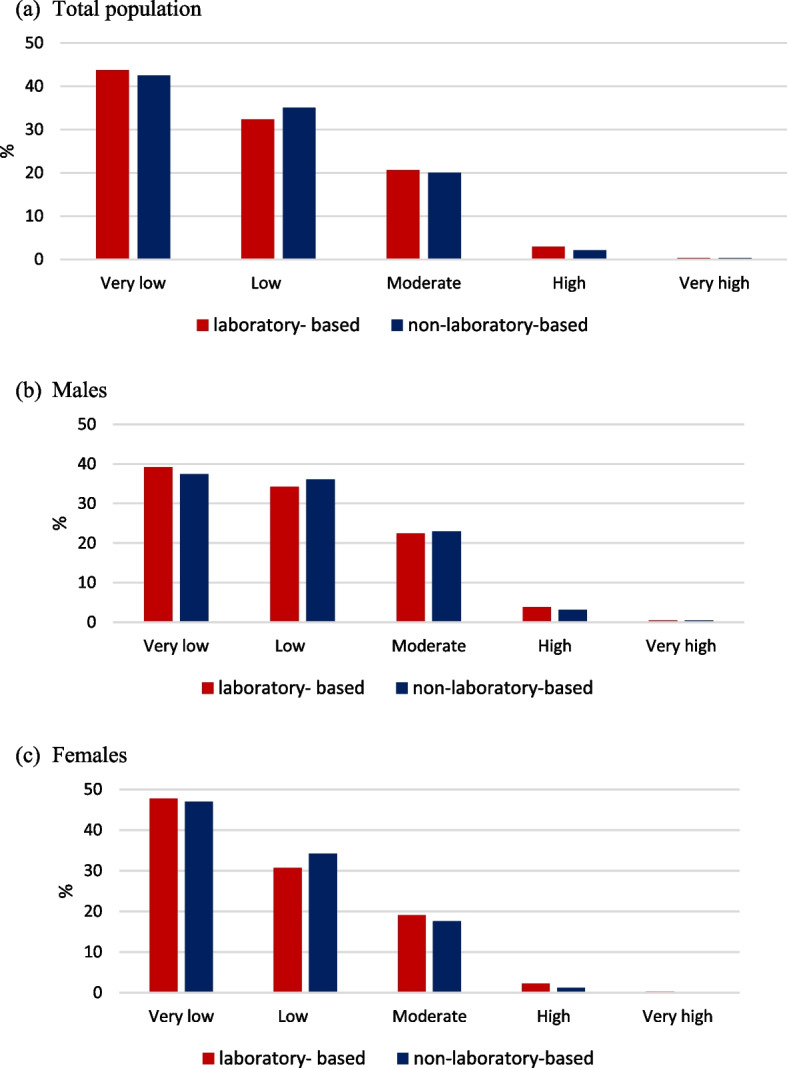
Percentage of cardiovascular risk classified according to laboratory-based and non-laboratory-based models. **a** Total population, **b** Males, **c** Females

The grouped risk of laboratory-based and non-laboratory-based models is shown in Fig. 1. The risk classification of these two models was very similar. In fact, in the laboratory-based model, the classification of very low, low, medium and high, and very high risk was 43.7%, 32.3%, 20.6%, 3.0% and 0.4%, respectively, and in the non-laboratory-based model, it was 42.5%, 35.1%, 20.0%, 2.1%, and 0.3%, respectively. Furthermore, in the laboratory-based model, 4.2% and 2.6% of males and females were at high and very high risk, respectively, and in the non-laboratory-based model, 3.7% and 1.3% of males and females were at high and very high risk.

### Mean differences in the laboratory-based and non-laboratory-based risk scores

The mean differences between laboratory-based and non-laboratory-based scores in total participants were 0.16% (95% CI: 0.11 to 0.21), which for males and females were 0.04% (95% CI: -0.03% to 0.12%) and 0.26% (95% CI: 0.19% to 0.34%), respectively.

Also, the mean difference was calculated for two age groups < 60 years and ≥ 60 years. The mean difference between the scores for those < 60 years was 0.12% (95% CI: 0.07 to 0.17) compared with 0.37% (95% CI: 0.19% to 0.56%) for those ≥ 60 years.

In the males, the mean difference of the scores was 0.08% (95% CI: -0.00 to 0.16) in those < 60 years old and -0.13% (95% CI: -0.37 to 0.11) in those ≥ 60 years old. In the females, the mean difference of the scores was 0.15% (95% CI: 0.08 to 0.22) in those < 60 years old and 0.84% (95% CI: 0.57 to 1.11) in those ≥ 60 years old.

### Bland–Altman plots / limits of agreement

**Fig. 2:**
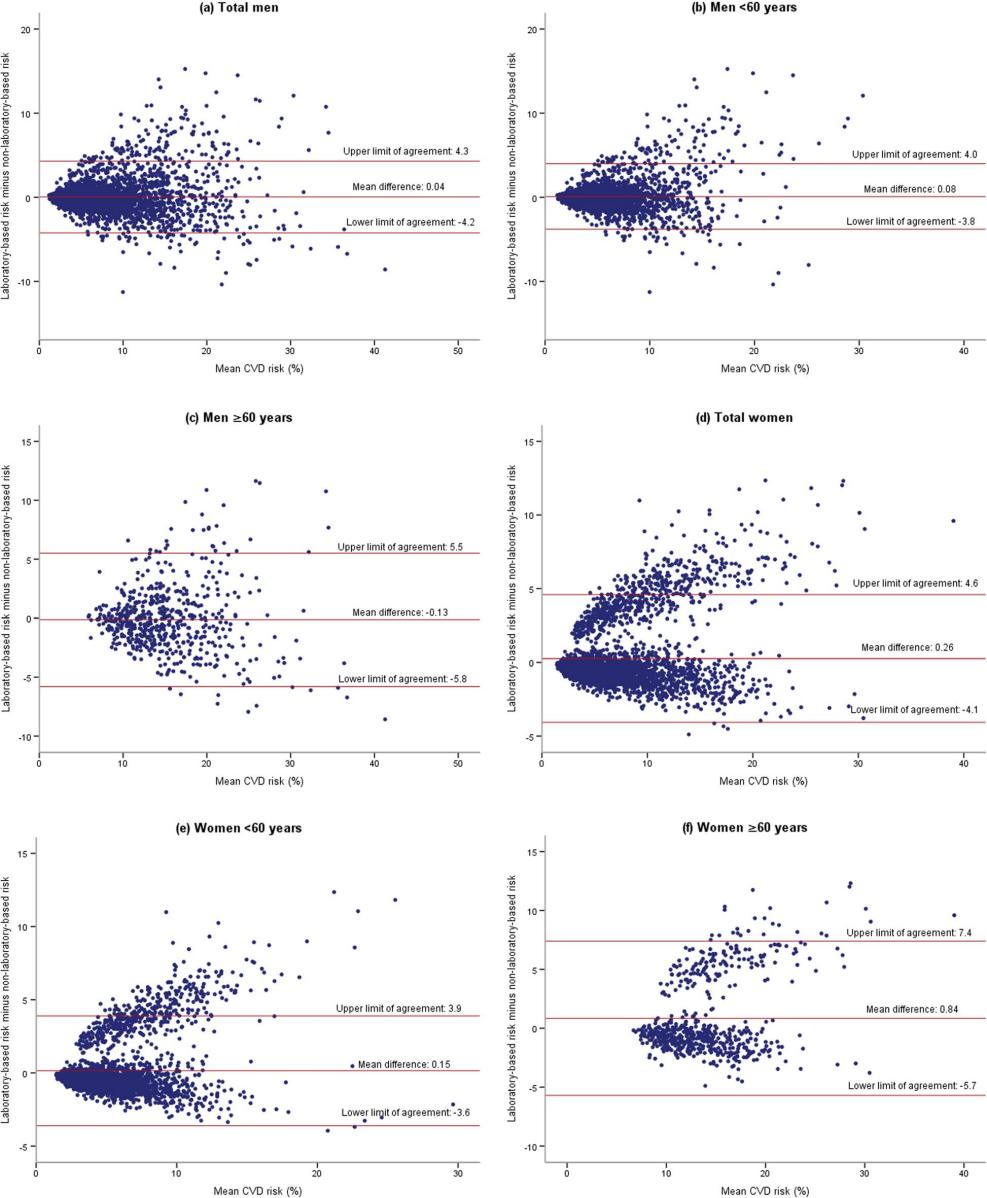
Bland–Altman plots showing the agreement between WHO laboratory-based and non-laboratory-based CVD risk scores for the predicted individual-level risk. The center horizontal line corresponds to the mean difference between the risk scores of laboratory-based and non-laboratory models. Upper and lower horizontal lines correspond to the 95% upper and lower limit of agreement, respectively. **a** Total men, **b** Men <60 years, **c** Men ≥ 60 years, **d** Total women, **e** Women <60 years, **f** Women ≥ 60 year

Bland–Altman plots to present agreement between the laboratory-based and non-laboratory-based scores for males and females by two age groups < 60 and ≥ 60 years old are shown in Fig. 2.

For all males, the limit of agreement was (-4.2% to 4.3%). Also, the limit of agreement was (-3.8% to 4.0%) and (-5.8% to 5.5%) for males < 60 years and ≥ 60 years, respectively. For all females, limit of agreement was (-4.1% to 4.6%). The limit of agreement was (-3.6% to 3.9%) and (-5.7% to 7.4%) for those < 60 years and ≥ 60 years, respectively. The limit of agreement was wider for those ≥ 60 years in comparison to < 60 years.

### Categorical agreement and kappa coefficients

In the whole population, the agreement between the two models was 79.0% (kappa = 0.68, standard error (SE) = 0.00). Categorical agreements between laboratory-based and non-laboratory-based models are shown for males in Table 3 and for females in Table 4. For males, the agreement between risk categories was 79.8% (kappa = 0.70, SE = 0.01). Furthermore, in the laboratory-based model, more males were in the high- and very high-risk group compared to the non-laboratory-based model (134 vs. 115). The agreement was better in males < 60 (79.9%, kappa = 0.67, SE = 0.01) than those ≥ 60 years old (79.7%, kappa = 0.59, SE = 0.03).

**Table 3:** Agreement between the laboratory-based and non-laboratory-based risk grouped in males by age grouped

Non-laboratory-based risk category	Laboratory-based risk category						Kappa
	**Very low**	**Low**	**Moderate**	**High**	**Very high**	**Total**	
**All males**							
Very low	1052	127	2	0	0	1181	
Low	182	844	110	3	0	1139	
Moderate	2	109	559	51	1	722	0.70
High	0	0	36	57	7	100	
Very high	0	0	0	9	6	15	
Total	1236	1080	707	120	14	3157	
**< 60 years old**							
Very low	1052	127	2	0	0	1181	
Low	182	784	91	3	0	1060	
Moderate	2	87	227	22	1	339	0.67
High	0	0	5	6	3	14	
Very high	0	0	0	0	0	0	
Total	1236	998	325	31	4	2594	
**≥ 60 years old **							
Very low	0	0	0	0	0	0	
Low	0	60	19	0	0	79	
Moderate	0	22	332	29	0	383	0.59
High	0	0	31	51	4	86	
Very high	0	0	0	9	6	15	
Total	0	82	382	89	10	563

**Table 4:** Agreement between the laboratory-based and non-laboratory-based risk grouped risk in females males by age grouped

Non-laboratory-based risk category	Laboratory-based risk category						Kappa
	**Very low**	**Low**	**Moderate**	**High**	**Very high**	**Total**	
**All females**							
Very low	1524	182	3	0	0	1709	
Low	212	831	202	0	0	1245	
Moderate	0	104	474	59	1	638	
High	0	0	14	20	9	43	0.66
Very high	0	0	0	3	1	4	
Total	1736	1117	693	82	11	3639	
** < 60 years old**							
Very low	1524	182	3	0	0	1709	
Low	212	755	171	0	0	1138	
Moderate	0	63	107	8	1	179	0.61
High	0	0	1	4	0	5	
Very high	0	0	0	1	0	1	
Total	1736	1000	282	13	1	3032	
≥ **60 years old**							
Very low	0	0	0	0	0	0	
Low	0	76	31	0	0	107	
Moderate	0	41	367	51	0	459	0.46
High	0	0	13	16	9	38	
Very high	0	0	0	2	1	3	
Total	0	117	411	69	10	607	

For males < 60 years old, the number of individuals in the high- and very-high risk group was higher in the laboratory-based model than in the non-laboratory-based model (35 vs. 14). However, in males ≥ 60 years old, the high- and very high- risk group was more in the non-laboratory-based model than in the laboratory-based model (101 vs. 99).

In females, the agreement of the two models was 78.3% (kappa = 0.66, SE = 0.01). More females were in the high- and very high-risk group in the laboratory-based model than in the non-laboratory-based model (93 vs. 44). The agreement in females < 60 years old was 78.8% (kappa = 0.61, SE = 0.01). In this age group, the number of females in the high-risk group was higher in the laboratory-based model than in the non-laboratory-based model (14 vs. 6). In females ≥ 60 years old, the agreement was 75.8% (kappa = 0.46, SE = 0.03) and more females were in the high- and very high- risk group in the laboratory-based model than in the non-laboratory-based model (79 vs. 41). The agreement was substantial in males and females < 60 years old but moderate in males and females ≥ 60 years.

### Performance of the non-laboratory-based model

Table 5 shows the performance of the non-laboratory-based model. At the risk threshold of 20%, the laboratory-based model is considered as the gold standard to detect high-risk individuals. For the non-laboratory model the sensitivity was higher in males compared to females (59.0% vs. 35.5%). Moreover, after age and sex grouping, the sensitivity was even higher in males aged ≥ 60 years than < 60 years (70.7% vs. 25.7%) but it was almost similar in females aged < 60 years and ≥ 60 years. According to the results, in males < 60 years, males ≥ 60 years, females < 60 years, and females ≥ 60 years MCC were 40%, 64%, 54%, and 44%, respectively.

**Table 5:** The clinical performance of the WHO non-laboratory-based model among 6796 adults in Fasa cohort study

	**Sensitivity**	**Specificity**	**PPV**	**NPV**	**MCC**
	**% (95%CI)**	**% (95%CI)**	**% (95%CI)**	**% (95%CI)**	
**WHO non-laboratory model**					
** Cut-off point of 20% for laboratory-based and non-laboratory-based models**					
**Male**					
All males	59.0 (50.6–67.3)	98.8 (98.4–99.2)	68.7 (60.2–77.2)	98.2 (97.7–98.7)	0.61
< 60 years old	25.7 (11.2–40.2)	99.8 (99.6–100)	64.3 (39.2–89.4)	99.0 (98.6–99.4)	0.40
≥ 60 years old	70.7 (61.7–79.7)	93.3 (91.1–95.6)	69.3 (60.3–78.3)	93.7 (91.5–95.9)	0.64
**Female**					
All females	35.5 (25.8–45.2)	99.6 (99.3–99.8)	70.2 (57.1–83.3)	98.3 (97.9–98.7)	0.49
< 60 years old	35.7 (10.6–60.8)	100 (99.9–100)	83.3 (53.5–100)	99.8 (99.5–99.9)	0.54
≥ 60 years old	35.4 (24.9 -45.9)	97.5 (96.2–98.9)	68.3 (54.1–82.5)	91.0 (88.6–93.4)	0.44
**Cut-off point of 20% for laboratory-based and 10% for non-laboratory-based model**					
**Male**					
All males	99.8 (95.2–1.00)	76.6 (75.1–78.1)	15.6 (13.2–18.1)	99.9 (99.7–100)	0.34
< 60 years old	91.4 (82.2–100)	87.4 (86.1–88.7)	9.0 (6.1–12.0)	99.9 (99.7–100)	0.27
≥ 60 years old	100 (100–100)	17.0 (13.6–20.4)	20.4 (16.9–24.1)	100 (100–100)	0.19
**Female**					
All females	100 (100–100)	83.2 (82.0–84.4)	13.5 (11.0–16.1)	100 (100–100)	0.33
< 60 years old	100 (100–100)	94.3 (93.5–95.1)	7.5 (3.7–11.0)	100 (100–100)	0.27
≥ 60 years old	100 (100–100)	19.9 (16.5–23.3)	15.7 (12.5–18.9)	100 (100–100)	0.18

*PPV* Positive predictive value, *NPV* Negative predictive value, *MCC* Matthews correlation coefficient

At the risk threshold of 10% in non-laboratory-based and 20% in laboratory-based models to detect high-risk individuals, the non-laboratory model had high sensitivity and negative predictive value of 100% in males ≥ 60 years, females < 60 and ≥ 60 years. In males < 60 years, sensitivity and negative predictive value were 91.4% and 99.9%, respectively. Also, In males < 60 years, males ≥ 60 years, females < 60 years, and females ≥ 60 years MCC were 27%, 19%, 27%, and 18%, respectively.

## Discussion

In this study, the agreement between the grouped risks of the two models was substantial. The limit of agreement was suitable for younger females and males.

Considering the fact that in LMICs, it may not be possible to estimate the 10-year risk of CVDs with a laboratory-based model which requires laboratory tests to determine the amount of cholesterol and the presence of diabetes. Due to the lack of resources in LMICs it is very crucial to determine the agreement of non-laboratory-based and laboratory-based models for estimating the 10-year risk of CVDs [29]. The agreement between laboratory and non-laboratory-based models was moderate in the global work convened by the 2019 WHO [12]. In Pars cohort study, Rezaei et al. reported a good agreement between laboratory-based and non-laboratory-based WHO models [23]. Jones et al. also revealed that there is a good agreement between BMI-based and cholesterol-based Framingham model [24]. Also, Jahangiry et al. estimated a good agreement between laboratory-based and office-based Globorisk equations in this population [18]. Guzman-Vilca et al. showed that there was the similarity between predicted CVD risk of the laboratory-based and non-laboratory-based WHO CVD risk models [30].

In the whole population, the mean difference between the risk scores of laboratory-based and non-laboratory models was negligible. This index was not only higher in females than males (0.26% vs. 0.04%) but also higher in the age group of ≥ 60 years old than < 60 years old (0.37% vs. 0.12%). According to classification by age and sex, the mean difference in females ≥ 60 years old was higher than males ≥ 60 years old (0.84% vs. -0.13%). In another study, the mean difference between laboratory-based and non-laboratory-based Framingham risk scores was 1.58% in males and 3.97% in female [17].

In this study, Bland–Altman plots were used to show the agreement between laboratory-based and non-laboratory-based risk scores at the individual-level. The agreement between the two risk scores was better in individuals < 60 years old than in ≥ 60 years old. It is worth noting that most studies have evaluated the agreement between different risk prediction models or the agreement between laboratory-based and non-laboratory-based Framingham risk scores, and we are not aware of any studies that have evaluated the agreement of laboratory-based and non-laboratory-based WHO models by Bland–Altman method. In another study, it was revealed that non-laboratory-based models can estimate the 10-year risk of CVDs with the same accuracy as laboratory-based models [31]. Rezaei et al. also showed that there is a very strong positive correlation between laboratory-based and non-laboratory-based WHO models in Pars cohort population [23]. Jahangiry et al. showed that a very strong direct correlation between laboratory-based and office-based Globorisk equations in Fasa cohort population [18]. It should be noted that, if the limit of the agreement obtained from the Bland–Altman analysis is within the pre-decided clinical agreement levels, the non-laboratory-based model can replace the laboratory-based model.

The results showed that there is a good agreement in the grouped risk of laboratory-based and non-laboratory-based models. The overall grouped risk agreement of the two models was substantial but the agreement was better in males than in females. The agreement was substantial in males and females < 60 years old. In those ≥ 60 years old, the agreement was moderate. In Sri Lanka, Mettananda et al. reported an almost perfect agreement between laboratory-based and non-laboratory-based models [32]. Another study indicated that the agreement between laboratory-based and non-laboratory-based WHO CVDs risk models was substantial for males < 60 years old, ≥ 60 years old and females ≥ 60 years old, but moderate for females > 60 years old [23]. Green et al. revealed that cholesterol-based and BMI-based Framingham models have 78.2% concordance in grouped risk [33]. The discrepancy among the results of different studies can be due to the difference in the study population, racial/ethnic differences in genetic predisposition, environment, cardiovascular risk factors and different risk prediction models [34]. Also, different WHO models may have different estimates, since most studies have used older WHO risk prediction models.

In the laboratory-based model, more females and males were in the high-risk group than in the non-laboratory-based model. There were 134 males in the high-risk group of the laboratory-based model but 115 males were in the non-laboratory-based model. Also, among females, 93 and 44 individuals were in the high-risk group in laboratory-based and non-laboratory-based models, respectively. Of course, it must be taken into consideration that the method of measuring risk factors can affect the risk score, but we should note that in the Fasa cohort, all measurements, including blood pressure and anthropometric indicators, were measured by trained people.

In the present study, an investigation was done to compare the validity of the non-laboratory-based model with laboratory-based model, which led to the following results. The non-laboratory-based model risk score has shown the sensitivity of 25.7%, 70.7%, 35.7%, and 35.4% for males < 60 years, males ≥ 60 years, females < 60 years and females ≥ 60 years, respectively at the risk threshold of 20%, respectively. Likewise, the specificity was > 90% in all age and sex groups. In 2023, Fahimfar et al. showed that the non-laboratory-based risk score has a high sensitivity and specificity [27]. Joseph et al. showed that precision of the non-laboratory-based risk scores were similar to laboratory-based models [13]. Pandya et al. suggested the non-laboratory-based model as a fruitful alternative for laboratory methods [35]. Rezaei et al. revealed that the non-laboratory-based model is able to categorize individuals almost identically to the laboratory-based model [23].

In this study, the performance of the test was different in sex and age groups. Sensitivity was higher in males ≥ 60 years. Age is one of the important CVD risk factors. There is evidences that the prevalence of CVD increases with age [36]. Also, Walli-Attaei et al. showed that men had a higher CVD risk factor burden [37]. This study found that sensitivity was higher in males ≥ 60 years. Recently, Leeflang et al. reported that the sensitivity and specificity of a test vary with the prevalence of a disease. This variation may be due to a mechanism such as patient spectrum that affects prevalence, sensitivity, and specificity. This effect was statistically significant for either sensitivity or specificity in 8 meta-analysis studies [38]. So, the prevalence of the low- and high-risk groups may affect the sensitivity and specificity. Of course, this should be confirmed in more studies. In addition, to increase the sensitivity of the non-laboratory model, a further analysis was conducted. To do so, the high-risk individuals are considered to be in non-laboratory-based with the risk threshold of 10% and in laboratory-based model with the risk threshold of 20%. In this case, a sensitivity of 100% in males ≥ 60 years, females aged < 60 and ≥ 60 years, and 91.4% in males aged < 60 years, was observed. Therefore, it was shown that for the screening program if the non-laboratory model used at the risk threshold of 10%, almost all individuals with a risk ≥ 20% in the laboratory model would be detectable. At the risk threshold of ≥ 10% in non-laboratory risk, only 26.6% of males and 18.8% of females needed lab measurements, and at the risk threshold of ≥ 20% in laboratory risk only 4.2% of males and 2.6% of females were in need of a change in lifestyle. And when needed, taking drugs as a treatment would decrease the risk of cardiovascular diseases, including heart attack and stroke in people who are more at risk of CVD. The results are almost similar to another study conducted in Iran. Fahimfar et al. showed that 36% of males and 28% of females with non-laboratory risk ≥ 10% need lab measurements, and about 6% of men and 4% of women with laboratory risk ≥ 20%, not only need lifestyle modification, but also will be in need of a more intensive intervention such as statin therapy [27]. The results showed that MCC was better in different sex and age groups at the risk threshold of ≥ 20% than at the risk threshold of ≥ 10% in non-laboratory-based model. However, the sensitivity of the non-laboratory-based model increases at the 10% cut-off point for the high-risk group. Therefore, more people who are at risk of CVD are identified. Consequently, the development or progression of the disease can be prevented with timely interventions. In this study at the risk threshold of 10%, the non-laboratory-based model which is simple and inexpensive, can be used for the screening programs in LMICs.

Laboratory-based and non-laboratory-based WHO models have eliminated the main obstacles on the way of global CVD prevention [20]. These models, especially non-laboratory-based models, are very important in LMICs that have limited resources. Since these models can be used for screening at a lower cost [12, 14]. Furthermore, they can be easily used in rural communities where laboratory tests and resources are limited [39]. According to the results of Dhana's study, in case of lack of resources, non-laboratory-based models can be used instead of laboratory-based models [40]. In this study, a good agreement between laboratory-based and non-laboratory-based was observed. Also, admissible sensitivity and specificity have been shown in the WHO non-laboratory-based model at the risk threshold of 10% compared with the laboratory model at the risk threshold of 20% in the population.

Considering that in most LMICs, public expenditures for health are insufficient and there is an excessive reliance on out-of-pocket payments as a source of health financing [41], it is possible to use the non-laboratory model instead of the laboratory-based model if needed. This is important especially in centers which do not have the necessary equipment to perform laboratory tests or in cases that are difficult to access centers that provide laboratory services. It is crucial to note that in this study, the risk threshold for high-risk individuals was suggested based on the non-laboratory-based model. However, CVD risk must be confirmed according to the laboratory-based model.

### Study strengths and limitations

According to the information we have, the present study is the first population-based study to evaluate the agreement between laboratory-based and non-laboratory-based WHO models with kappa statistics and Bland–Altman plots in such a large population. Owing to the large sample size, it is likely that the findings of this study can be generalized to the general population of Iran. This cross-sectional analysis was conducted using the baseline data of a cohort study, and further prospective analyses are needed to examine the validity of laboratory-based and non-laboratory-based models.

## Conclusion

In the total population, substantial agreement was observed between the risk groups of laboratory-based and non-laboratory-based models. At the risk threshold of 20% for laboratory-based and 10% for non-laboratory-based model, sensitivity and specificity of the WHO non-laboratory-based were acceptable. Therefore, in countries with limited resources and insufficient investment in the healthcare sector, the non-laboratory-based model can be used in primary health centers for screening and risk assessment programs. Using these models helps individuals be aware of their CVDs risk as accurately as the laboratory-based model, yet at a lower cost. Additionally, it helps physicians make the best clinical decision. As these models perform risk assessment like laboratory-based models, they can ultimately lead to the prevention of morbidity and mortality due to CVDs and thus, reduce the burden of CVDs.

## Data Availability

In this study the dataset analyzed are available from the corresponding author upon reasonable request.
